# Comparison of Staging Systems of Hepatocellular Carcinoma

**DOI:** 10.1155/2011/818217

**Published:** 2011-06-27

**Authors:** Yongyut Sirivatanauksorn, Chutwichai Tovikkai

**Affiliations:** HPB and Transplantation Unit, Division of General Surgery, Department of Surgery, Faculty of Medicine Siriraj Hospital, Mahidol University, Bangkok 10700, Thailand

## Abstract

Many staging systems of hepatocellular carcinoma (HCC) were established; however, there is no consensus on which is proper in predicting prognosis. This study aims to evaluate various commonly used staging systems of HCC. Patients who underwent surgery during 2001–2007 were included. All patient data were retrospectively staged using six staging systems, that are American Joint Committee on Cancer (AJCC) Tumour-Node-Metastasis (TNM), Okuda staging, Cancer of the Liver Italian Program (CLIP), Barcelona Clinic Liver Cancer (BCLC), Chinese University Prognostic Index (CUPI), and Japan Integrated Staging (JIS). Child-Pugh classification was also evaluated. The staging systems were compared by mean of overall and disease-free survival. Total of 99 patient data were enrolled in the analyses. All staging systems except Okuda were significant in determining overall survival in univariate analyses. In multivariate analyses, TNM and Child-Pugh demonstrated better predictive power for overall survival. In terms of disease-free survival, univariate analyses revealed that TNM, CLIP, BCLC, CUPI, and JIS were significant, and TNM was the best predictive staging system in multivariate analyses. In our study, TNM and Child-Pugh are the representative systems in predicting survival of HCC patients who undergo surgical resection. Moreover, they are practical and easily assessable in clinical practice.

## 1. Background

Hepatocellular carcinoma (HCC) is the most common primary malignancy of liver and one of the most common malignancies especially in Eastern and Southeastern Asia. The most important risk factors of HCC are chronic hepatitis B, C and cirrhosis. 

In malignancy diseases, staging system is important because it defines prognosis and is a guiding tool for treatment options and also a research tool for comparison between different groups and trials [1]. American Joint Committee on Cancer (AJCC) uses tumour-node-metastasis (TNM) system as staging system for many malignancy diseases to predict prognosis [2]. Nevertheless, in HCC, AJCC/TNM system fails to stratify patients adequately with respect to prognosis because TNM system evaluates only tumour extension. Since the remnant liver function is another important factor to prognosis of patients with HCC beside tumour burden; therefore, the staging system for HCC should include these both factors [3]. 

Staging systems that include liver function status were first proposed by Okuda et al. in 1985 based on study of 850 HCC patients [4]. This Okuda staging system was consisted of tumour load, ascites, albumin, and bilirubin. It was accepted and used widely as an improved classification system for HCC. Since the introduction of this staging system about two decades ago, when most HCC cases were diagnosed in the advanced stage, there was much progression in diagnostic and therapeutic tools for patients with HCC. Most patients are now diagnosed in less extensive disease. The Okuda staging system does not properly identify patients who may be suitable for certain therapeutic interventions [5]. Recently many staging systems for HCC were proposed, they were known as newer scores. In 1998, the Cancer of the Liver Italian Program (CLIP) investigators proposed the CLIP score that is based on Child-Pugh grading, distribution of tumour(s), alpha-fetoprotein (AFP) level, and portal vein thrombosis [6]. This scoring system was validated prospectively in 196 patients and showed greater predictive power than Okuda staging system [7]. In 1999, the Barcelona Clinic Liver Cancer (BCLC) staging classification for HCC was proposed by Llovet et al. in the aim of defining prognosis and treatment strategies [8]. It stratified tumours into four risk groups and proposed different planning of treatment for each group. Recently, it was validated prospectively in 195 patients in Italy and demonstrated a better prognostic ability than AJCC/TNM 2002 system in surgical patients [3]. In Asia, there were also some proposed staging systems as they claimed that the natural history might be different in different places around the world. The Chinese University Prognostic Index (CUPI) for HCC was identified in Hong Kong on the basis of a cohort of 926 Chinese patients in 2002 [9]. It combines the conventional TNM system with some factors of liver function and tumour load. The authors reported that the CUPI was better than TNM, Okuda, and CLIP staging system in predicting survival. In 2003, the Japan Integrated Staging (JIS) score was proposed by Kudo et al. [10]. It is based on new adapted TNM system proposed by the Liver Cancer Study Group of Japan (LCSGJ) and Child-Pugh grading. In this study, it showed that JIS was superior to CLIP score in selecting the best prognosis patients group.

Though many staging systems for HCC were introduced around the world, there is no consensus on which the best in predicting prognosis and selecting patients into different treatment planning [1]. The aim of this study was to evaluate and compare the various commonly used staging systems for HCC in term of predicting prognosis of the Thai patients who underwent surgical resection.

## 2. Materials and Methods

This study was retrospective cohort study. Patients who were admitted for surgical resection in Department of Surgery Siriraj Hospital during January 2001 and June 2007 were included in the study. Medical records were reviewed. Diagnosis of HCC was confirmed by histopathological report or by clinical and radiological finding that serum alpha-feto protein (AFP) level more than 200 ng/dl associated with radiological investigation is suggestive of HCC. The patients with incomplete information after reviewing medical records and who were lost to followup were also excluded from the study.

The data was reviewed and collected in terms of geographic data including ECOG performance status [11], clinical presentation, tumour characteristics both pathological and radiological, cause and severity of cirrhosis, biochemical and immunological data (e.g., viral hepatitis status), and operative details.

All patient data were retrospectively staged using the six staging systems, that is, American Joint Committee on Cancer (AJCC) tumour-node-metastasis (TNM) 2002 system, Okuda staging system, Cancer of the Liver Italian Program (CLIP) scoring system, Barcelona Clinic Liver Cancer (BCLC) staging system, Chinese University Prognostic Index (CUPI), and Japan Integrated Staging (JIS). Child-Pugh classification for cirrhosis was also evaluated.

All patients were followed up to December 2007. Last status at followup, recurrence, and mortality were recorded. Disease-free survival was calculated from date of operation to date of recurrence. Overall survival was calculated from date of diagnosis to date of death.

In data analysis, comparison between two groups of continuous data was analysed using the unpaired *t*-test and the Mann-Whitney *U* test. Relationship between nominal and ordinal data was analysed using the Chi-squared test or the Fisher's exact test. Univariate survival analyses of each staging system were calculated using the Kaplan-Meier method and compared by means of the log rank test. A stratified Cox proportional hazard regression model was used for multivariate analyses. Statistical significance is defined as a *P* value of ≤ 0.05. The SPSS version 10.0 software package was used for this statistical analysis.

## 3. Results

Total of 181 patient data were reviewed, and 82 patients of these were excluded. There were incomplete data for evaluation in 15 patients, and 67 patients were lost to followup. Therefore 99 patients were enrolled in the analyses. Mean age of the patients was 57.6 years with range from twenty-three to eighty-one years. There were male more than female patients at the ratio about three to one (M : F = 75 : 24).

In terms of aetiology of cirrhosis, there were chronic hepatitis B 47 patients (47.5%), chronic hepatitis C 11 patients (11.1%), chronic hepatitis B and C coinfection 1 patient (1.0%), alcoholic cirrhosis 4 patients (4.0%), cryptogenic cirrhosis 1 patient (1.0%), and unspecified 25 patients (25.2%).

In surgical resection type, there were right hepatectomy 17 patients (17.2%), left hepatectomy 9 patients (9.1%), anatomical segmental resection 22 patients (22.2%), nonanatomical wedge resection 38 patients (38.4%), and other operations (e.g., liver biopsy, liver packing) 13 patients (13.1%). Thirty days postoperative mortality was 5.1 percent. There were three patients died from sepsis, one from ruptured HCC, and one from postoperative bleeding. Follow-up time was up to one hundred and ten months, with mean at 26 months.

**Figure 1:**
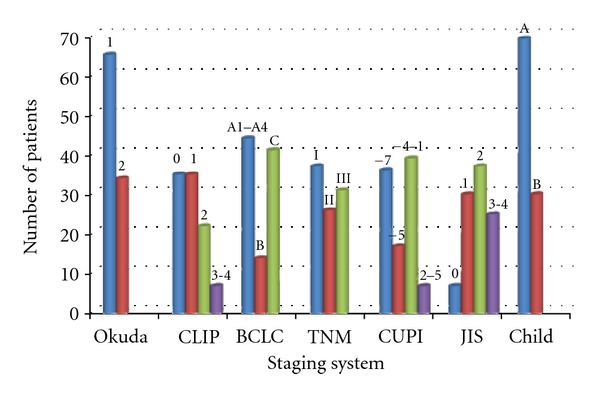
Patients data categorized using six staging systems and Child-Pugh classification.

The patient data was categorized using six staging systems and also Child-Pugh classification, as described in Figure 1. There were 65 patients in Okuda stage 1, 34 patients in Okuda stage 2, and none in Okuda stage 3. Thirty-five patients had CLIP score of 0, 35 patients had CLIP score of 1, 22 patients had CLIP score of 2, and 5 patients had CLIP score of 4. Forty-four patients were categorized in BCLC A1–A4, 14 patients in BCLC B, 41 patients in BCLC C, and none in BCLC D. In TNM staging system, there were 37 patients in stage I, 26 patients in stage II, 19 patients in stage IIIa, 10 patients in stage IIIb, 2 patients in stage IIIc, and none in stage IV. In CUPI staging system, 36 patients got score of −7, 17 patients got −5, 6 patients got −4, 14 patients got −3, 5 patients got −2, 7 patients got −1, 6 patients got 0, 1 patient got 1, 4 patients got 2, and 3 patients got score of 5. Seven patients had JIS 0, 30 patients had JIS 1, 37 patients had JIS 2, 23 patients had JIS 3, and 2 patients had JIS 4. Sixty-nine patients had cirrhosis Child A, 30 patients had cirrhosis Child B, and none had Child C.

**Figure 2:**
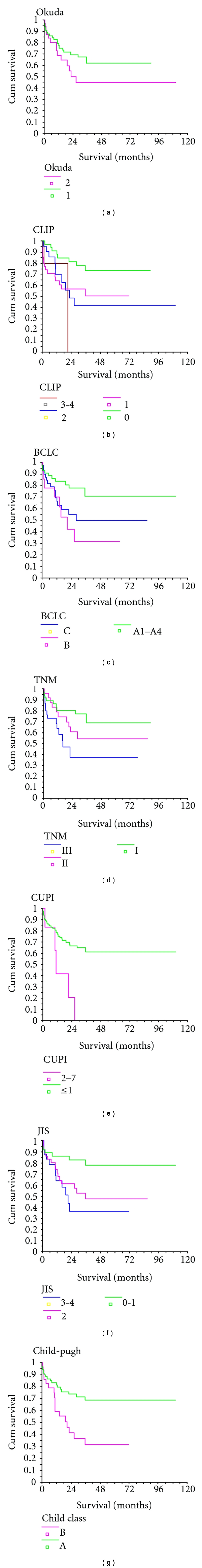
Kaplan-Meier survival curves of overall survival.

In overall survival, all of the staging systems except Okuda staging system were significant in the univariate analyses (Table 1) and enrolled in multivariate analysis. In multivariate analyses, TNM staging system and Child-Pugh classification demonstrated better predictive power for overall survival comparing with CLIP, BCLC, CUPI, JIS systems with *P* value of 0.020 and 0.021, respectively (Table 2, Figure 2).

**Figure 3:**
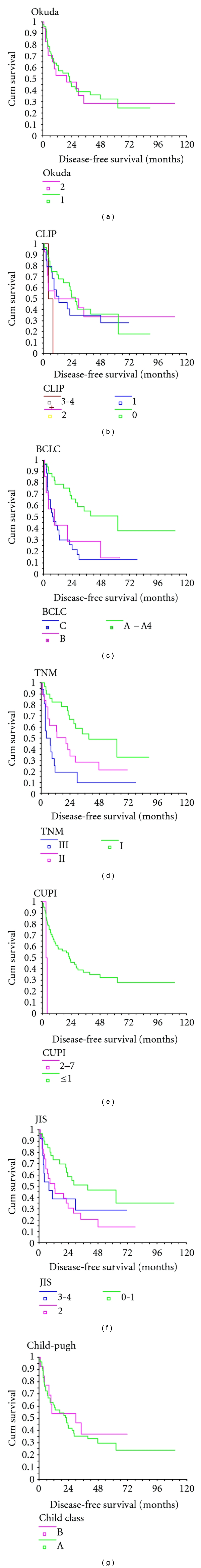
Kaplan-Meier survival curves of disease-free survival.

In terms of disease-free survival, univariate analyses demonstrated that CLIP, BCLC, TNM, CUPI, JIS staging were significant with *P* value of 0.0289, 0.0003, 0.0001, 0.0128, 0.0157, respectively (Table 3). In multivariate analyses, TNM was shown to be the best predictive staging system with *P* value of 0.001 (Table 4, Figure 3).

## 4. Discussion

Staging system is very important in every malignant disease. It does not only predict prognosis but also provides plan of management. It was an important tool to categorize and compare patients in research study. Most of all, there was only one commonly used staging system for each malignancy, but not in HCC. HCC is one of the distinctive malignancies because it has two pathological changes in one disease. Firstly, it is the tumour change from hepatocytes itself and the other is cirrhosis, chronic inflammation, and fibrotic change of liver parenchyma. Most of HCCs are arising on top of cirrhosis. Cirrhosis and liver function status are very important factors in determining plan of treatment especially surgical resection. 

Nowadays, there are many staging systems proposed worldwide. Some are simple and easy to remember such as Okuda staging system. Some are more complicated but claimed to have more predictive power. There are many publications comparing these staging systems, but until now there is no consensus on which is the best prognostic staging system for HCC [12–17]. This study would be the first study about staging systems of HCC in Thai patients who underwent surgical resection.

In general, staging systems of HCC have divided into 2 categories, clinical staging system and pathological staging system. The clinical staging systems usually define patients initially at pretreatment state and include clinical, radiological, and laboratory data. Nevertheless, they would not include pathological factors that would be obtained after surgical resection. The pathological staging systems almost always include data from pathological reports that have to be confirmed after surgery, and they will provide more informative data in terms of predicting survival. However, they can only be applied to patients who have undergone surgery [18]. Thus in surgical patients, staging systems that include data from pathological report should comprise more discriminating power to predict survival.

The applicability of staging systems of HCC is also dependent on the selection of treatment. The predictive power may be different in each group of patients. For example, the best staging system for HCC patient who underwent surgery might not be suitable for patients with advanced disease who only received supportive care. In a cohort of 2010 Taiwanese patients, the Tokyo staging system was the best in predicting survival for patients receiving surgical resection or transarterial chemoembolization while CLIP scoring system was the most suitable in predicting survival in HCC patients receiving chemotherapy or supportive care [14]. In patients who underwent surgical resection, there are many studies showing that the most suitable staging system for surgical HCC patients was TNM [15, 19], CLIP [20, 21], or JIS [22].

Furthermore, different staging systems have different predictive power for HCC patient in different area of the world, roughly the East and the West. That could be from the difference in tumour biology and also the aetiology of liver cirrhosis. In Eastern countries, studies from China, Korea, and Taiwan demonstrated that either TNM [15, 19, 23] or CLIP [20, 24] had better predictive power than others, while many studies from Japan favoured JIS as the best staging system [22, 25, 26]. In contrary, most studies from Western countries suggested that either BCLC [3, 27, 28] or CLIP [29, 30] had superior discriminatory power.

Most of patients in this study were distributed in earlier stage in both tumour biology and liver parenchymal or cirrhotic change. There was no patient in most advanced stage such as in Okuda stage 3, TNM stage IV, and BCLC stage D. This result was caused by the selection of study population which included the patients who were candidates for surgery and certainly these patients would have early stage of disease. Patients who had advanced disease were not suitable for surgery. The selection of such patients cause the limitation of the application of the results of this study to only the surgical candidates. 

In overall survival, the most predictive staging systems in this study are AJCC/TNM staging system and Child-Pugh classification. AJCC/TNM staging system contains only tumour factor and does not include liver function status. It has the best predictive power in both overall survival and recurrence. On the other hand, Child-Pugh classification is a good prognostic factor of liver function in cirrhosis and used widely. It can also predict overall survival, but not recurrence. When combined together, these two systems are still the best systems in predicting survival of patients with HCC especially in the group of the surgical candidates. They are also practical, easy to remember and to use. This study is the one among other studies that emphasizes pathological staging system for patients under surgery.

AJCC/TNM staging system was also validated in patients who underwent liver transplantation for HCC. Vauthey et al. conducted multicenter study in US and Europe comparing many staging systems, including, AJCC/TNM, CLIP, JIS, and BCLC in terms of predicting survival after liver transplantation for HCC. The AJCC/TNM staging system had the best stratification of prognosis for patients who underwent liver transplantation. Combining with previous studies in patients who underwent surgical resection, the authors proposed that AJCC/TNM staging system provided a uniform evaluation of prognosis in HCC patients who underwent surgery including both surgical resection and liver transplantation [31]. The American-Hepato-Pancreato-Biliary Association (APHPBA) and the AJCC Consensus Conference also recommended the use of the sixth edition of AJCC/TNM staging system for surgical patients after surgical resection and liver transplantation [18]. 

There are still some drawbacks of AJCC/TNM staging system in the very early stage of HCC. Even in early HCC such as tumour size less than 5 cm, no major vascular invasion, node negative, no metastasis, that is, T1N0M0, there were still some factors that affect survival outcome of the patients. Nathan et al. studied a cohort of early stage HCC patients and found tumour size, even in tumour size ≤5 cm, multifocality, and microvascular invasion as important factors that influenced the patients' survival. They proposed new staging system called Early HCC prognostic score that was summarized from each point for each factor [32]. This score was validated in multicenter trial compared to many staging systems including Okuda, International Hepato-Pancreato-Biliary Association (IHPBA) staging system, CLIP, BCLC, JIS, and AJCC/TNM. Early HCC prognostic score and AJCC/TNM showed good stratification of patients who underwent surgical resection or liver transplantation, while others did not. In fact, early HCC prognostic score was superior to AJCC/TNM for predicting survival of early HCC patients [33]. 

In conclusion, though there are many new staging systems, TNM staging system and Child-Pugh classification are the proposed prognostic staging systems in determining survival of postsurgical resection HCC patients in our study. Although the result suggests using these two systems combined together as a staging system in HCC especially in the surgical candidate patients because they are practical, easily assessable, and simple applications in the clinical practice, the authors consider that some other staging systems such as Tokyo staging system and early HCC prognostic score would have a major role in predicting the survival. Therefore, further study should be performed in the large population including the nonsurgical patients and should put on some new scoring systems such as IHPBA or Early HCC prognostic scoring systems in the analysis.

## Figures and Tables

**Table 1:** Univariate analyses of overall survival data.

Staging system	*P *value
Okuda	0.1844
CLIP	0.0213
BCLC	0.0117
TNM	0.0148
CUPI	0.0093
JIS	0.0070
Child-Pugh	0.0039

**Table 2:** Multivariate analyses of overall survival data.

Staging system	Multivariate analysis		
	Hazard ratio	95% CI	*P* value
Okuda	—	—	n/a
CLIP	—	—	0.517
BCLC	—	—	0.323
TNM	2.702	1.168–6.251	0.020
CUPI	—	—	0.182
JIS	—	—	0.253
Child-Pugh	2.238	1.132–4.424	0.021

**Table 3:** Univariate analyses of disease-free survival data.

Staging system	*P* value
Okuda	0.8569
CLIP	0.0289
BCLC	0.0003
TNM	0.0001
CUPI	0.0128
JIS	0.0157
Child-Pugh	0.5728

**Table 4:** Multivariate analyses of disease-free survival data.

Staging system	Multivariate analysis		
	Hazard ratio	95% CI	*P* value
Okuda	—	—	n/a
CLIP	—	—	0.053
BCLC	—	—	0.789
TNM	4.193	1.950–9.015	0.001
CUPI	—	—	0.088
JIS	—	—	0.160
Child-Pugh	—	—	n/a
